# Anticancer Effects of Sinulariolide-Conjugated Hyaluronan Nanoparticles on Lung Adenocarcinoma Cells

**DOI:** 10.3390/molecules21030297

**Published:** 2016-03-02

**Authors:** Kuan Yin Hsiao, Yi-Jhen Wu, Zi Nong Liu, Chin Wen Chuang, Han Hsiang Huang, Shyh Ming Kuo

**Affiliations:** 1 Department of Biomedical Engineering, College of Medicine, I-Shou University, Kaohsiung City 82445, Taiwan; kireihsiao@pie.com.tw (K.Y.H.); purple19900104@yahoo.com.tw (Y.-J.W.); jeff821231@gmail.com (Z.N.L.); 2 Department of Electric Engineering, College of Electrical and Information Engineering, I-Shou University, Kaohsiung City 84001, Taiwan; cwchang@isu.edu.tw; 3 Department of Veterinary Medicine, College of Agriculture, National Chiayi University, Chiayi City 60054, Taiwan

**Keywords:** sinulariolide, aggregate, nanoparticle, hyaluronan, lung adenocarcinoma cells

## Abstract

Lung cancer is one of the most clinically challenging malignant diseases worldwide. Sinulariolide (SNL), extracted from the farmed coral species *Sinularia flexibilis*, has been used for suppressing malignant cells. For developing anticancer therapeutic agents, we aimed to find an alternative for non-small cell lung cancer treatment by using SNL as the target drug. We investigated the SNL bioactivity on A549 lung cancer cells by conjugating SNL with hyaluronan nanoparticles to form HA/SNL aggregates by using a high-voltage electrostatic field system. SNL was toxic on A549 cells with an IC_50_ of 75 µg/mL. The anticancer effects of HA/SNL aggregates were assessed through cell viability assay, apoptosis assays, cell cycle analyses, and western blotting. The size of HA/SNL aggregates was approximately 33–77 nm in diameter with a thin continuous layer after aggregating numerous HA nanoparticles. Flow cytometric analysis revealed that the HA/SNL aggregate-induced apoptosis was more effective at a lower SNL dose of 25 µg/mL than pure SNL. Western blotting indicated that caspases-3, -8, and -9 and Bcl-xL and Bax played crucial roles in the apoptotic signal transduction pathway. In summary, HA/SNL aggregates exerted stronger anticancer effects on A549 cells than did pure SNL via mitochondria-related pathways.

## 1. Introduction

Lung cancer is one of the most common malignancies worldwide. It is the leading cause of cancer-related mortalities, accounting for more than 1.5 million deaths worldwide in 2012 [1]. According to the World Health Organization, lung cancer can be classified into small cell lung cancer (SCLC) and non-small cell lung cancer (NSCLC) [2], NSCLC being the more prevalent. NSCLC is mostly diagnosed at an advanced stage (either locally advanced or metastatic disease), and late-stage lung cancer is extremely difficult to treat. The malignant behavior of NSCLC is caused by different driver mutations, which may include alterations in the epidermal growth factor receptor (EGFR) signaling pathway. In the past years, it has been found that activating mutations in exons 19 or 21 of EGFR in NSCLC are associated with increased sensitivity to EGFR tyrosine kinase inhibitors (TKIs) such as gefitinib and erlotinib. Most patients with NSCLC are recommended to undergo either curative or palliative treatment with TKIs. Treatment of advanced NSCLC has changed dramatically. However, the present survival rate for patients with NSCLC is still unsatisfactory, and the analyzed 5-year overall survival rate for lung cancer (all stages) is only 15% [3]. Therefore, it is urgent to discover more effective agents against lung cancer for possible higher response rate, longer survival time, and better therapeutic strategies.

New drugs are developed globally by finding and extracting therapeutic materials from natural sources. Some extracts from plants, such as paclitaxel and docetaxel, have been clinically and widely used as anticancer drugs [4,5]. Given that water is more abundant on earth than is land and that the oceans are rich and diverse in natural resources, one can reasonably assume that potential anticancer agents can be derived from marine materials. Natural compounds extracted from marine sponges and soft corals have been found to possess bioactive effects such as anti-inflammatory, anti-microbial and anti-tumor activities. Sinulariolide (SNL), an active component isolated from the cultured soft coral *Sinularia flexbilis*, has been demonstrated to inhibit malignant cells *in vitro*. Recent studies have demonstrated that SNL can promote apoptosis in hepatocellular carcinoma HA22T and melanoma A375 cells [6,7]. The potential anti-proliferative and apoptosis-induced effects of SNL on human bladder cells *in vitro* have also been reported [8]. Despite its anticancer activity, SNL has some limitations, such as low bioavailability and poor hydrophilicity. Furthermore, high drug doses are often required for exerting a therapeutic effect on cancer tissues, which causes undesirable effects on the surrounding healthy tissues. To solve the aforementioned problem, we developed well-adapted drug-delivery systems, such as nanoparticles, which reduce the amount of administered drugs. Nanomedicine is a division of nanotechnology in which the performance of nanoparticles is applied to medicine and healthcare areas. One of its essential medical applications is to drug delivery system. The size of nanostructured materials is ranged from 1 to 100 nm and these nanovehicles have high loading capacity and specific targeting to tumor cells due to their size effect and intracellular uptake. Nanoparticles used for drug delivery are submicron (<1 µm) colloidal particles prepared from biocompatible and biodegradable materials. Recent improvements in particle technology have enabled customizing nanoparticle-based drug-delivery systems for specific target tissues, pharmacokinetic profiles, and administration routes [9]. The achievements and challenges of nanoparticle-based drug delivery have been reported. We previously demonstrated that dihydroartemisinin-aggregated gelatin and hyaluronan (HA) nanoparticles enhance apoptosis in A549 cells [10]. HA, a natural glycosaminoglycan, is biodegradable, biocompatible, nontoxic, hydrophilic, and nonimmunogenic. An exogenous HA administration arrests tumor spreading [11]. Major concerns have recently been voiced over HA in the developing field of drug-delivery systems; HA is used in various drug-delivery methods, such as encapsulation in various types of nanoassemblies as a ligand for preparing nanoplatforms for actively targeting drugs, genes, and diagnostic agents [12]. We have previously fabricated the biopolymeric nanoparticles using an electrostatic field system (EFS) manner in an aqueous-phase environment as the drugs/compounds with anti-tumor activities have been successfully aggregated by the biopolymeric nanoparticles in the EFS [13]. In this study the natural marine compound SNL extracted from a sponge *Sinularia flexbilis* was aggregated by biopolymeric HA nanoparticles using the EFS method and the anti-tumor and apoptosis-induced effects of pure SNL as well as aggregated SNL were further investigated and examined using MTT assay, migration assay, flow cytometric analysis and western blot *in vitro*. According to our review of the relevant literature, this is the first *in vitro* study to evaluate the effects of SNL on A549 cells.

## 2. Results and Discussion

### 2.1. Characteristics of HA/SNL Aggregates

**Figure 1:**
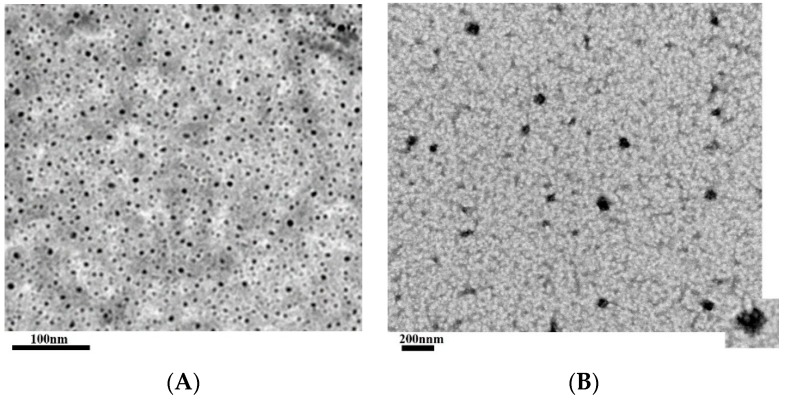
TEM images of (**A**) HA nanoparticles and (**B**) HA/SNL25 aggregates.

HA nanoparticles prepared using the EFS were well dispersed in solution and exhibited a spherical shape with a mean diameter ranging from approximately 5–7 nm. When the hydrophobic SNL drug was incorporated into the HA nanoparticle production process, the HA nanoparticles would be glued together by the SNL which dissolved in DMSO. Consequently, the hydrophilic domain of SNL would bind itself onto the hydrophilic sites or region of HA nanoparticles, and the hydrophobic region of SNL bound to the hydrophobic regions of HA nanoparticles, forming a thin continuous layer and thus aggregating the individual HA nanoparticle with irregular shapes and increasing sizes into an approximately 33–77 nm scale. The TEM images of HA nanoparticles and HA/SNL aggregates are shown in Figure 1.

**Figure 2:**
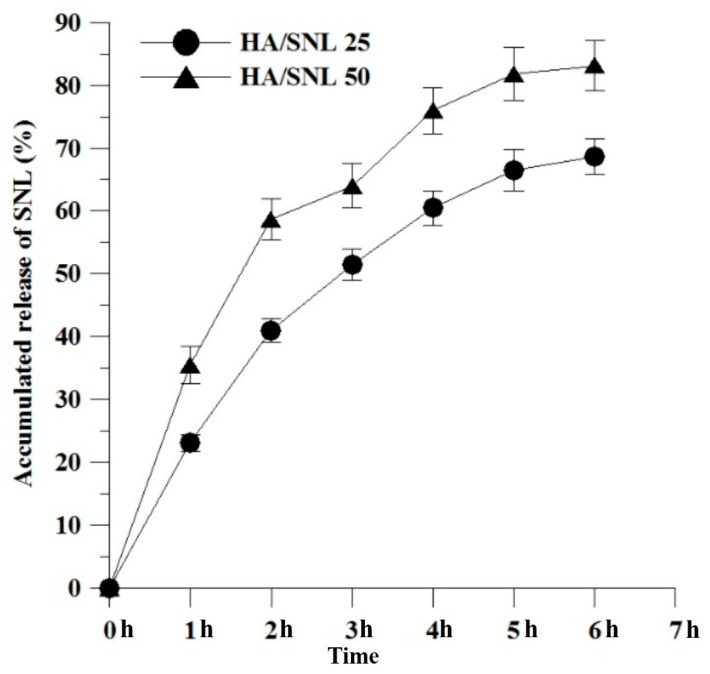
*In vitro* release profiles of SNL from HA/SNL25 and HA/SNL50 aggregates in PBS (pH 7.4).

The incorporation efficiency of SNL 25 and SNL 50 within HA nanoparticles were approximately 74% and 81%. As shown in TEM images, the SNA was glued by HA nanoparticles and exhibited an irregular and moderate packed morphology, which might be due to the hydrophobic/hydrophilic interactions between the HA nanoparticles and SNL drug. These interactions yielded a relative high incorporation efficiency of SNL drug. However, the formation mechanism and the production parameter about these aggregates would be examined in our future study. The release profile of SNL from HA/SNL aggregates with an initial burst release (approximates 25% for HA/SNL/25 and 33% for HA/SNL 50) during the 1-h of incubation was presented in Figure 2. Approximately 67% SNL was released from HA/SNL25 aggregates and about 81 % SNL was released from HA/SNL 50 aggregates after 6-h incubation. The rapid release of SNL form aggregates might be attributed by moderate packed morphology of HA/SNL aggregates. 

### 2.2. Hyaluronan Nanoparticle Cytotoxicity Assay

**Figure 3:**
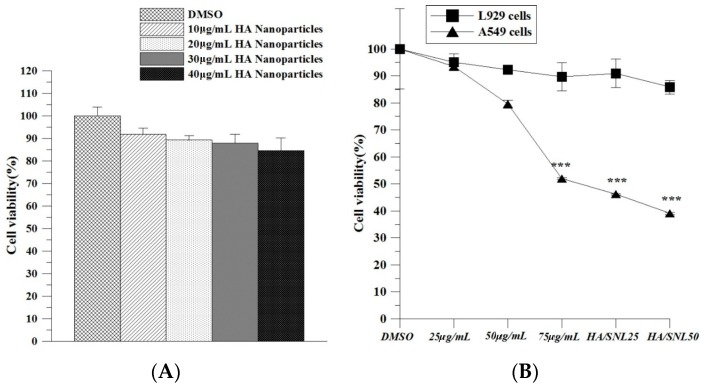
Cytotoxicity of HA nanoparticles, SNL and HA/SNL aggregates on L929 cells and A549 cells. (**A**) Viabilities of A549 cells treated with various HA nanoparticles concentrations; (**B**) Cell viability dose-response curves for L929 cells and A549 cells treated with SNL and HA/SNL aggregates over 24 h. The significant differences are based on comparisons with the L929 cells and A549 cells. *** *p* < 0.001.

We examined the HA nanoparticles’ cytotoxicity prior to planned experiments for identifying any undesired toxic effects imposed on the cultured cells. A549 cells were treated with various concentrations of HA nanoparticles, and no evident decrease in cell viability was observed (Figure 3). The results demonstrated that HA nanoparticles were nontoxic.

### 2.3. IC_50_ of SNL and in Vitro Cell Viability

**Figure 4:**
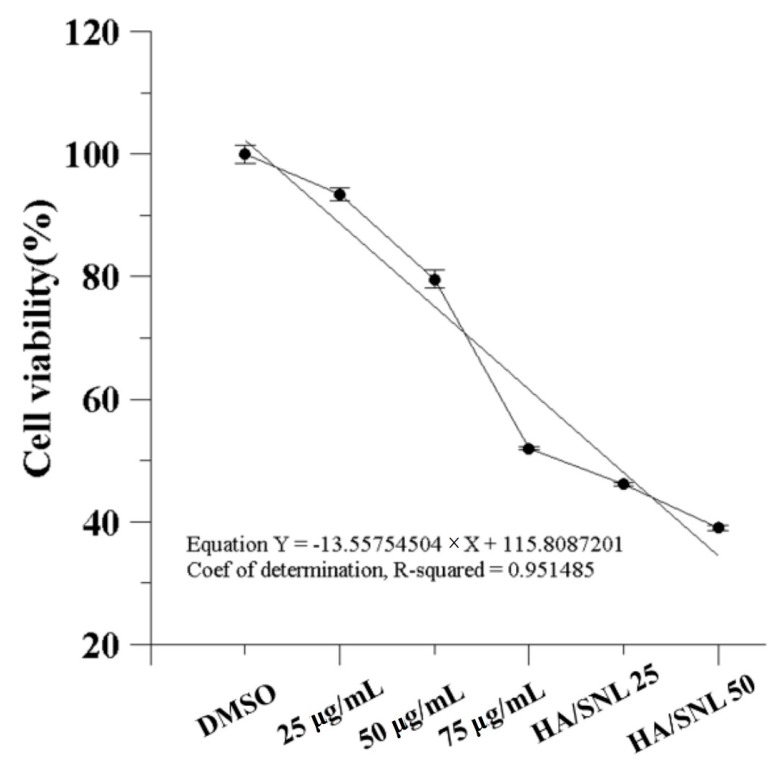
Effect of SNL and HA/SNL aggregates on A549 cell viability. Cell viability remained 50% (IC_50_) when treated with 75 µg/mL pure SNL.

**Figure 5:**
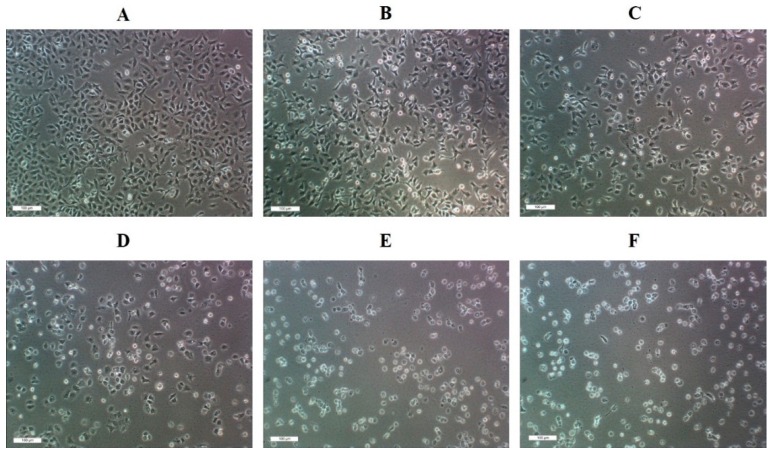
Morphology of A549 cells after a 24-h treatment with DMSO (**A**); 25, 50, and 75 µg/mL SNL (**B**, **C**, and **D**, respectively), and HA/SNL aggregates 25 (**E**) and 50 (**F**).

IC_50_ is the drug concentration causing 50% inhibition of the desired activity. An MTT assay (Figure 4) revealed that the IC_50_ of SNL was approximately 75 µg/mL. DMSO treatment on A549 cells (control group) did not inhibit cell proliferation. By contrast, cell viability significantly decreased after a 24-h treatment with 75 µg/mL SNL, HA/SNL 25, and HA/SNL 50 each, indicating that HA/SNL aggregates strengthened the anticancer effect, with a considerable decrease in the anticancer and cell viability effect on A549 cells treated with SNL alone. Dead A549 cells showed distinctive apoptotic morphological characteristics, including shrinkage, frilled membranes, and detachment from the culture plate surface (Figure 5), particularly in the HA/SNL aggregate groups.

### 2.4. Live/dead Assay of A549 Cells

**Figure 6:**
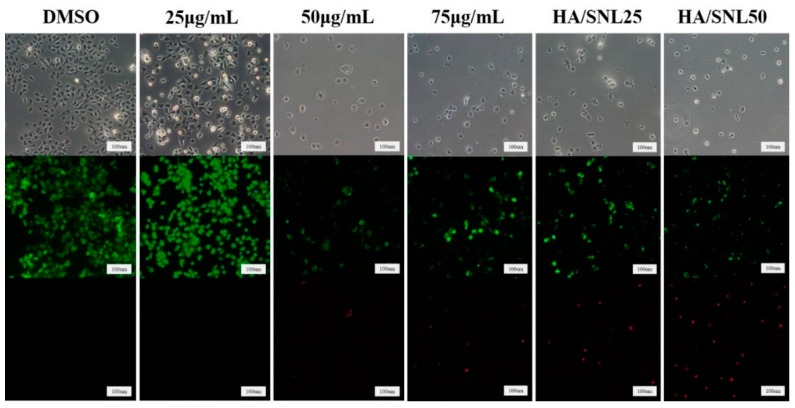
Live/dead assay of A549 cells after a 24-h treatment with SNL or HA/SNL aggregates. The fluorescence micrographs show the live (green) and dead (red) A549 cells after treatment.

A live/dead cell assay clarified cell viability after treatments with SNL and HA/SNL aggregates. Figure 6 shows viable (green) and dead (red) A549 cells after a 24-h treatment with SNL and HA/SNL aggregates. A549 cells treated with DMSO and 25 µg/mL SNL exhibited no evident dead cells; however, dead cells appeared at high SNL concentrations. The higher amount of dead cells in the HA/SNL aggregate groups indicates that the aggregates induced more apoptosis.

### 2.5. Antimigratory Effect of SNL and HA/SNL Aggregates on A549 Cells

**Figure 7:**
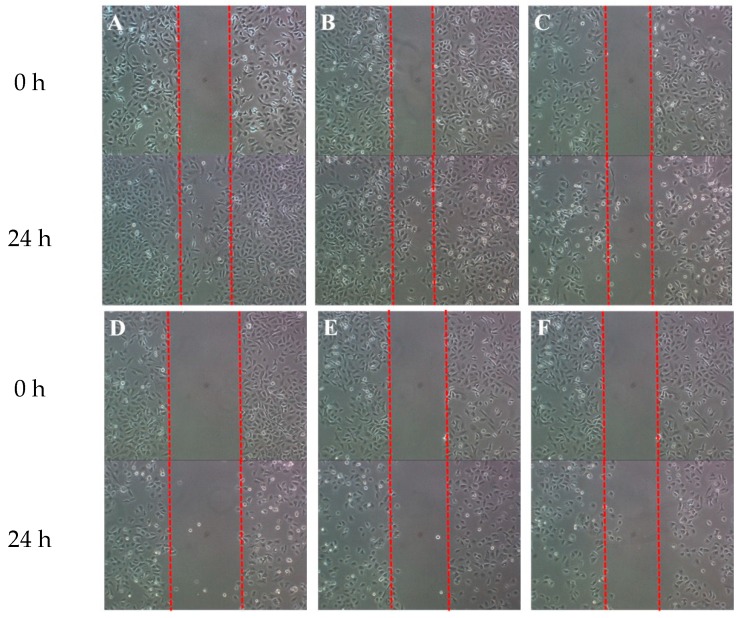
Wound healing assay of A549 cells treated with (**A**) DMSO, control group; (**B**) SNL 25 µg/mL; (**C**) SNL 50 µg/mL; (**D**) SNL 75 µg/mL; (**E**) HA/SNL aggregates 25; and (**F**) HA/SNL aggregates 50.

The antimigratory effects of SNL and HA/SNL aggregates on A549 cells were assessed through a wound healing assay, which is commonly used for investigating cell migration and its underlying biology. Figure 7 shows that increasing the SNL concentration reduced A549 cell migration in the artificial wound. The antimigratory effects of HA/SNL 25 and 50 were nearly identical to those observed with 75 µg/mL of SNL alone, indicating that HA/SNL aggregates have antimigratory effect at a lesser SNL dose.

### 2.6. Apoptosis Assay through Flow Cytometry

**Figure 8:**
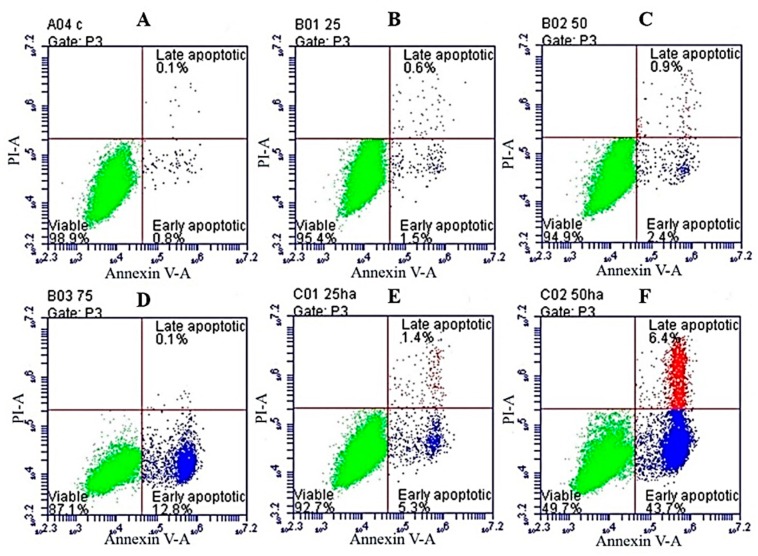

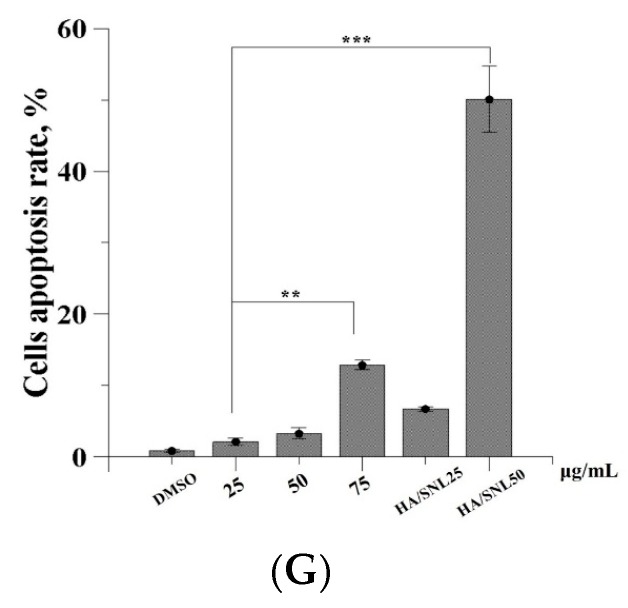
Annexin V-FITC/PI-based apoptosis profiles of A549 cells after a 24-h SNL and HA/SNL aggregates treatment. (**A**) DMSO; (**B**) 25 µg/mL; (**C**) 50 µg/mL; (**D**) 75 µg/mL; (**E**) HA/SNL 25; (**F**) HA/SNL 50; and (**G**) total apoptotic rate of cells treated with DMSO, pure SNL, and HA/SNL aggregates (sum of early apoptosis and late apoptosis). Red: live cells, green: apoptotic cells, blue: background (** *p* < 0.01, *** *p* < 0.001).

To examine the apoptotic effects of HA/SNL aggregates on A549 cells, annexin V-FITC/PI staining was used; the fluorescence was quantified through flow cytometry. The control group cells displayed a low background staining with either annexin V-FITC or PI (Figure 8A). The number of annexin V-FITC- and PI-positive cells gradually increased in cells incubated with 25, 50, and 75 µg/mL SNL (Figure 8B–D, respectively).

The cells treated with HA/SNL aggregates showed a considerable increase in annexin V-FITC- and PI-positive staining for early and late apoptosis, respectively. The HA/SNL 50 group specifically yielded approximately 50.1% apoptotic cells with significant differences compared with the aforementioned SNL groups (Figure 8E–G). The apoptosis rates are listed in Table 1.

**Figure 9:**
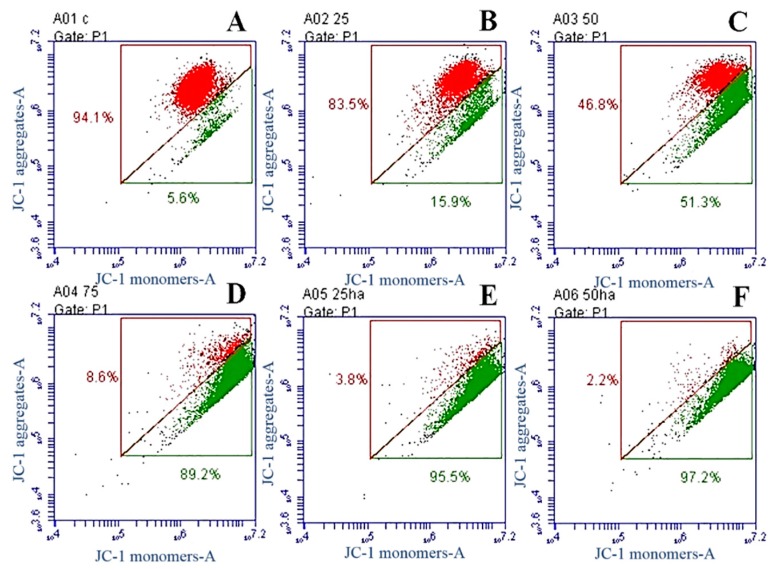

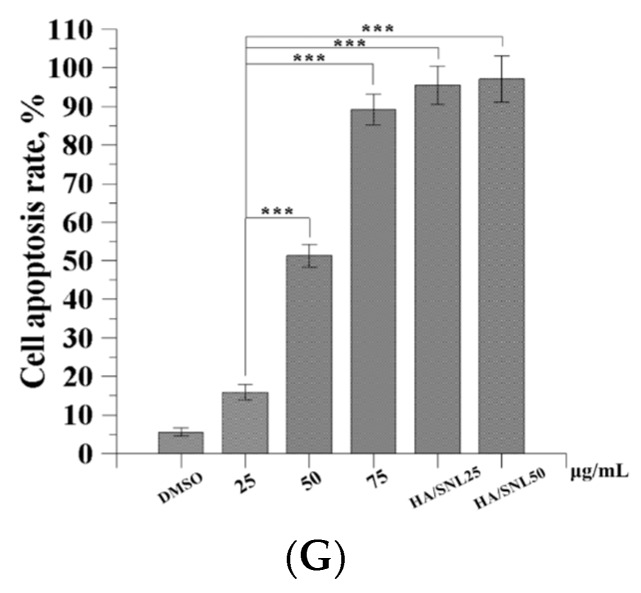
JC-1 staining-based apoptosis profile of A549 cells after a 24-h SNL and HA/SNL aggregates treatment. (**A**) DMSO;(**B**) 25 µg/mL; (**C**) 50 µg/mL; (**D**) 75 µg/mL; (**E**) HA/SNL 25; (**F**) HA/SNL 50; and (**G**) the total apoptotic rate of cells treated with DMSO, pure SNL, and HA/SNL aggregates (sum of early apoptosis and late apoptosis). Red: live cells, green: apoptotic cells (*** *p* < 0.001).

ΔΨm was calculated to confirm whether mitochondria-related apoptosis occurred because of SNL or HA/SNL aggregate treatments. A549 cells showed an increase in apoptosis after treatments with 25 and 50 µg/mL SNL (Figure 9B,C, respectively). A considerable increase in apoptosis was observed for 75 µg/mL SNL and HA/SNL aggregate groups (Figure 9D–F), where the HA/SNL 50 treatment caused approximately 97.2% apoptosis. Results suggest that HA/SNL aggregates with less SNL concentrations caused a ΔΨm loss nearly identical to that observed in cells treated with high SNL doses and significantly differed from that in the other groups (Figure 9G).

### 2.7. Cell Cycle Analysis

**Figure 10:**
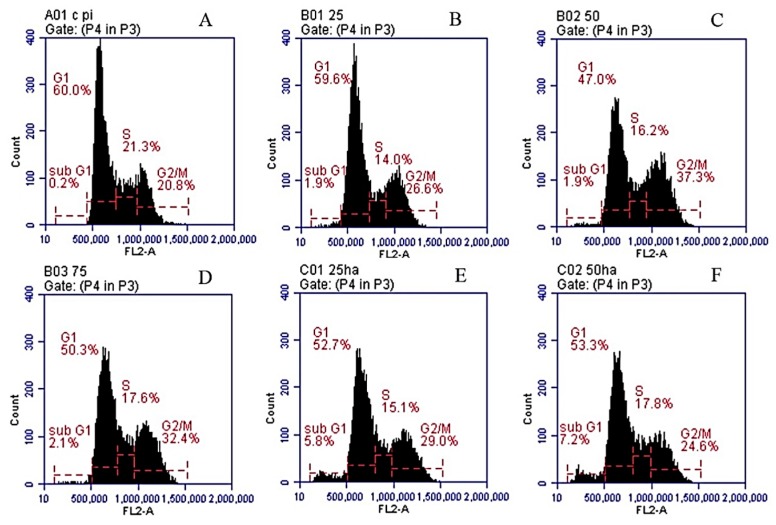

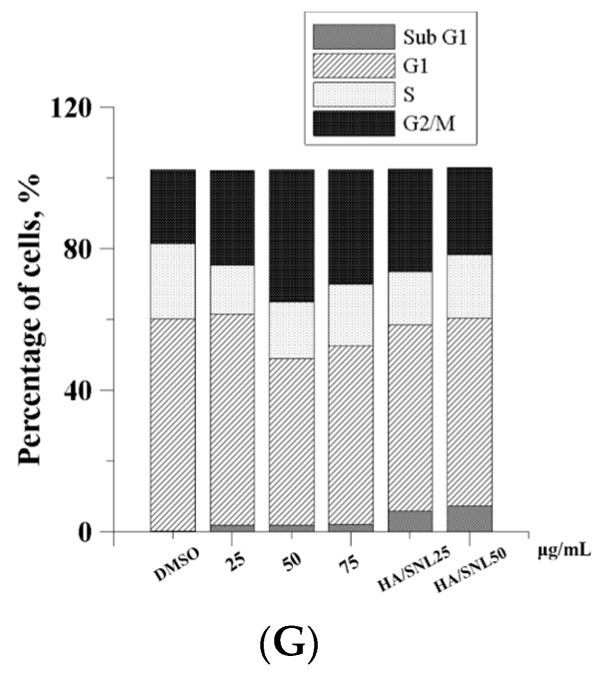
PI staining-based cell cycle analysis of A549 cells treated with DMSO, SNL, and HA/SNL aggregates. (**A**) DMSO; (**B**) 25 µg/mL; (**C**) 50 µg/mL; (**D**) 75 µg/mL; (**E**) HA/SNL 25; (**F**) HA/SNL 50; and (**G**) a summarized cell distribution representing mean ± SE (see Table 2).

Cell cycle progression was analyzed to evaluate the antiproliferative effects of SNL and HA/SNL aggregates on A549 cells. Cells treated with SNL and HA/SNL aggregates exhibited a high proportion of G2/M phase, indicating that the SNL treatment arrested cells in G2/M phase (Figure 10). A high proportion of sub-G1 phase was also observed in cells treated with SNL and HA/SNL aggregates. The sub-G1 phase percentage was 3.5% for the 75 µg/mL SNL group, higher than that in the control group (0.1%). HA/SNL 25 and 50 groups showed more increase in sub-G1 phase percentage (7.4% and 9.2%, respectively), which is indicative of enhanced apoptosis. Cell cycle data indicated that SNL and HA/SNL aggregates not only have the ability to enhance early apoptosis but also cause cell cycle arrest in the G2/M phase in A549 cells. The phase distribution for A549 cells treated with SNL and HA/SNL aggregates is shown in Table 2.

### 2.8. Western Blot Analysis

**Figure 11:**
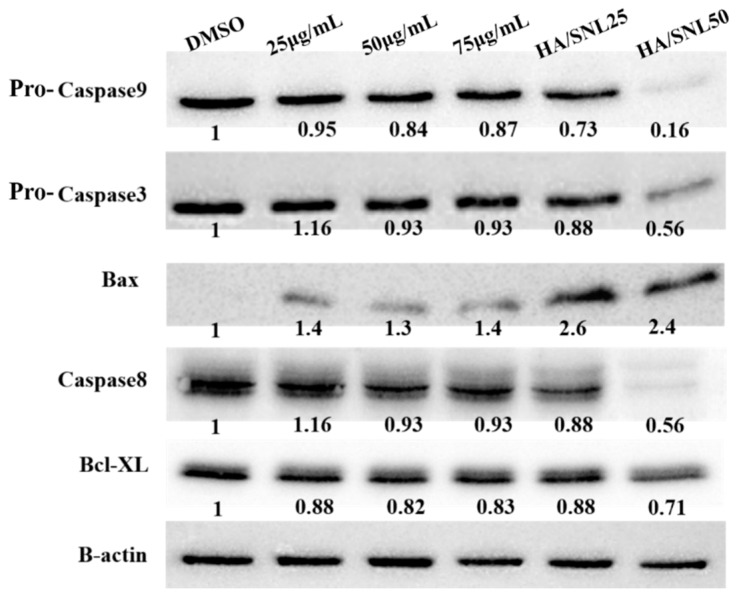
Western blot analysis for detecting apoptosis-related proteins after a 12-h treatment with DMSO, SNL, and HA/SNL aggregates.

To investigate whether the observed apoptosis involved caspase activation, a western blot analysis was performed on cell extracts collected after the treatments (Figure 11). Levels of apoptosis-related proteins, pro-caspase-3, -8, and -9, Bax, and Bcl-xL, expressed in A549 cells were evaluated through western blot analyses. Caspases-3, -8, and -9 are situated at pivotal junctions in the apoptotic pathways. A 12-h incubation with HA/SNL aggregates reduced protein levels of pro-caspase-3, -8, and -9, and the antiapoptotic factor Bcl-xL, indicating that HA/SNL aggregates suppress A549 cell proliferation through apoptosis. The level of Bax (mitochondria-related apoptotic marker) increased in HA/SNL aggregate groups. These results suggest that HA/SNL aggregates induce apoptosis via the mitochondria-related apoptotic pathway. The western blot analysis of HA/SNL aggregate groups showed considerable decreases in pro-caspase-3, -8, and -9, and Bcl-xL levels and a high Bax level compared with those observed for 25, 50, and 75 µg/mL SNL. The aforementioned increases and decrease reveal that the increasing SNL concentration is involved in elevated apoptosis induction.

Many chemotherapeutic and target agents have been identified against lung cancer, but their effectiveness is unsatisfactory, one reason being that nonspecific reactions of anticancer agents evidently damage healthy tissues [14]. Therefore, new drugs and specific drug-delivery systems have been explored to reduce the side effects. The present research focused on an active compound extracted from soft corals for identifying new anticancer therapeutic agents. Natural compounds extracted from marine soft corals and sponges exert activities against various tumor cell types such as melanoma cells, leukemia cells, hepatocellular carcinoma cells and bladder cancer cells [15,16]. Chen *et al.* [6] reported that SNL dose-dependently suppressed proliferation in hepatoma HA22T cells and induced both early and late apoptosis. In addition, SNL possessed evident inhibitory effects against A2058 melanoma cells and human bladder carcinoma cells [8,17]. Nanotechnology is a prospective field that offers substantial improvement for therapeutic interventions against malignancies presently challenging oncologists. In cancer treatment, nanotechnology has been applied in cancer diagnosis, therapy, and prevention [8]. Nanomedicine can potentially revolutionize cancer treatment, and one of the most noticeable models is malignant melanoma [18]. Melanoma is a highly aggressive malignancy; it is difficult to treat using traditional treatment strategies. Nanotechnology provides a platform for encapsulating drugs, increasing cytotoxities, more precise targeting of the tumor, and delivering small interfering RNAs for melanoma treatment, all of which have together yielded promising outcomes. In the development of nanoparticle preparation, a new method to produce biopolymeric nanoparticles by using EFS in an aqueous-phase environment has been developed and experimented. In our previous study, we have aggregated potential anti-tumor compounds including dihydroartemisinin and curcumin with biodegradable and biocompatible nanosized HA, chitosan and gelatin particles using EFS to successfully reinforce their anti-cancer activities against non-small cell lung cancer cells [19,20]. We have also used EFS to demonstrate that HA undergoes a self-assembly process to form nanoparticles with a narrow range of particle sizes; thus, facilitating incorporation of hydrophobic drugs in HA nanoparticles to form aggregates [8,11]. In this study, SNL is a hydrophobic compound; therefore we used EFS to prepare HA nanoparticles and HA/SNL aggregates.

Aggregation/encapsulation of nanoparticles including HA enhances anti-tumor effects of potential compounds and drugs. HA is an anionic, nonsulfated glycosaminoglycan that is critical in cell growth, proliferation, and adhesion. As a drug delivery system carrier, HA has numerous functional groups obtainable for conjugation. Appropriate nanoparticle sources, particle size, shape, charge, and surface chemistry should be determined by considering the desired bioactivity of the encapsulating drugs. In this study, we chose HA, which is biodegradable and biocompatible, as the carrier. The usage of HA nanoparticles in human lung cancer A549 cells has been investigated. Rivkin *et al*. [21] coated paclitaxel, a widely used antitumor agent for lung cancer, with HA and reported that the aggregate was as potent as a four-fold high paclitaxel dose. The current study examined the anti-lung tumor effects and apoptotic pathways of SNL extracted from the marine sponge and further investigated the improvement and mechanisms of the anti-tumor effects of SNL after HA nanoparticle aggregation using MTT assay, migration assay, flow cytometric analysis, JC-1 staining, western blotting analysis, and wound healing assay.

Our primary objective was to evaluate the apoptosis-induced anticancer effects of SNL on A549 cells and to determine whether these apoptotic-induced effects could be enhanced further through an HA/SNL aggregate treatment. Our results indicated that SNL exerted anticancer effects, such as proliferation arrest, apoptosis, and cell cycle arrest, on A549 cells. The degree of early apoptosis caused by SNL was dose dependent (1.5%, 2.4%, and 12.8% for 25, 50, and 75 µg/mL, respectively); however, SNL did not significantly interfere with late apoptosis. Furthermore, SNL caused cell cycle arrest in sub-G1 phase, where 3.5% A549 cells were detected in sub-G1 phase after a 24-h treatment with 75 µg/mL SNL. The sub-G1 phase arrest is evidently caused by apoptosis. 

Nanoparticles are similar in sizes to the characteristic cellular components and can efficiently penetrate into living cells through endocytosis rather than through passive diffusion. Endocytosis is an energy-dependent process through which cells internalize ions and biomolecules [22]. Improvements in pharmacokinetic properties of nanoparticles are attributable to endocytosis through the protection of the encapsulated drugs from the cell membrane pump efflux process, which usually removes the internalized drugs [23]. The endocytosis pathways are typically classified into clathrin- and caveolae-mediated endocytosis, phagocytosis, macropinocytosis, and pinocytosis. Because nanoparticles are favorable potential drug carriers, understanding their endocytosis mechanisms is crucial.

This study showed that SNL cytotoxic effects on A549 cells were correlated with the drug concentration, indicating that large doses would be required for achieving the optimal treatment result. However, large doses are mostly accompanied with severe side effects, which may cause morbidities and mortalities clinically. Hence, developing suitable drug carriers and drug-delivery systems that improve the drug’s pharmacotherapeutical effect is essential to enhance the treatment utilization rate and to reduce the amount of prescribed doses. In this study, our data demonstrated that the anticancer proliferative effects of SNL were enhanced after encapsulating SNL within HA nanoparticles and even low doses sufficiently achieved effective pharmacotherapeutic results (Figure 4). These data suggest that the aggregation of HA nanoparticles is capable of increasing the cytotoxic and apoptosis-inducing effects of SNL and may thus lower the prescribed drug doses and reduce the possible side effects if the medicine used clinically. 

Our flow cytometric analyses demonstrated that cells treated with HA/SNL aggregates exhibited a higher ratio of early apoptosis (43.7%) compared with cells treated with 50 µg/mL SNL alone (2.4%) (Figure 8). Furthermore, a higher proportion of sub-G1 phase arrest was observed for HA/SNL 25 (7.4%) and HA/SNL 50 (9.2%) aggregate groups compared with 25 (1.9%) and 50 (3.4%) µg/mL SNL groups, which is suggestive of enhanced apoptosis. These results suggested that hydrophilic HA nanoparticle conjugation notably increased the SNL bioactivity. Furthermore, our previous study demonstrated that HA nanoparticles could yield more apoptosis in A549 cells, when conjugated with curcumin and dihydroartemisinin [8,24]. One study presented that HA nanoparticles could enhance the efficacy of the conjugated anticancer drug doxorubicin against drug-sensitive and drug-resistant human ovarian cancer cells *in vitro* and ovarian tumor models in nude mice *in vivo* [25]. HA exhibits several properties that facilitate its use as a successful drug carrier. This water soluble, nonimmunogenic polysaccharide has multiple functional groups available for chemical conjugation [26]. Furthermore, HA is a major CD44 ligand and can be used for targeting cells expressing CD44 [27]. In the current study, we utilized the HA polymer to enhance the targeted drug delivery into cancer cells rather than toxically exposing the surrounding healthy cells.

The most effective solution to treat cancer is its eradication. Many anticancer therapies involve tumor cell death through apoptosis and necrosis [28,29]. In the current study, the mitochondrial-associated, caspase-dependent pathways of pure SNL and SNL/HA aggregates-induced apoptosis were examined by JC-1 staining and western blot. The JC-1 staining results showed that pure SNL from 25 to 75 μg/mL gradually elevated rates of JC-1 monomers (green), implicating an increase of cytochrome c into cytosol. Additionally, JC-1 staining showed that HA/SNL aggregates caused more ΔΨm loss than that caused by SNL. Furthermore, treatment with SNL/HA nanoparticles at 25 μg/mL produced about 6-fold elevation in JC-1 green monomer rate. The western blot analysis revealed the involvement of caspases-3 and -9 and Bax. The JC-1, Annexin V/PI staining and western blotting data indicated that the mechanism of HA/SNL-induced cell apoptosis is mainly mitochondria related, and HA nanoparticle aggregation is capable of increasing the SNL-induced apoptosis. Induction of apoptosis is directed by intrinsic or extrinsic pathways. Mitochondria are vital in the intrinsic pathway, and are regulated by Bcl-2 families through the mitochondrial outer membrane permeabilization (MOMP) process. The caspase protease activity is essential for apoptosis; once active, caspases cleave various proteins leading to a rapid cell death [30]. MOMP results in activation of caspase 9, which activates caspase 3 initiating apoptosis. Bax is a pro-apoptotic Bcl-2-family protein present in the cytosol and translocates to mitochondria upon apoptosis induction. Recently, Bax has been shown to induce cytochrome C release and caspase activation [31], and this release was associated with ΔΨm [32]. From these results combined with the aforementioned transmembane transport pathways, it can rationally be derived that pure hydrophobic SNL enters lung cancer cells through a simple diffusion method, whereas HA/SNL nanoparticle aggregates are internalized via clathrin- or caveolae-mediated endocytosis pathway. Furthermore, HA/SNL aggregates might separate into pure SNL and HA nanopartilces components after the internalization, where pure SNL induces apoptosis, which is consistent with the data presented in current and previous studies; HA nanoparticles further promote and strengthen apoptosis via the mitochondria-related pathway resulting in the summation or even synergism of cytotoxic and apoptosis-inducing effects on lung cancer cells. Correlating evidence can be found in MTT or live/dead staining, flow cytometric, and JC-1 staining results in the present study. Therefore, our data demonstrated that both SNL and HA/SNL aggregates could induce lung cancer cell apoptosis via the mitochondria-related and caspase-dependent pathways. The current results also suggest that HA nanoparticle-aggregation promisingly benefits the anti-cancer effects of potential and current chemotherapeutics.

## 3. Experimental Section

### 3.1. Production of Hyaluronan/Sinulariolide Aggregates

We built an electrostatic field system (EFS) for producing the HA/SNL aggregates [13]. Briefly, EFS was created using two parallel copper plates (18 × 6 cm) as the distance between the plates is 2 cm. The electric field strength between the plates was provided by a DC power supply and a functional generator was used to control the strength. The EFS was constructed in a thermal chamber for temperature control and safety purposes. SNL was dissolved in dimethyl sulfoxide (DMSO) to prepare a stock solution (25 mg/mL). Then, 1 mL of a HA solution (0.2 mg/mL) was vigorously mixed with various amounts of SNL. The HA/SNL mixture was poured into a plastic petri dish placed in the center of two plate electrodes, and such parameters as temperature (25 °C), applied electrostatic field strength (5 kV/cm), reaction time (1 h), and cross-linking reagent were well-controlled during production. After 1 h of treatment, the prepared HA/SNL aggregates were initially placed on formvar-coated copper grids. These grids were negatively stained with 2% phosphotungstic acid and air-dried. The air-dried grids were examined and analyzed using a Tecnai G2 20 S-Twin transmission electron microscope (TEM; FEI, Hillsboro, OR, USA). The size of the aggregates was estimated through TEM by randomly sampling approximately 100 individual aggregates, which minimized any selection bias. HA/SNL aggregates containing 25 and 50 µg/mL SNL are denoted as HA/SNL 25 and HA/SNL 50, respectively.

### 3.2. SNL Incorporation Efficiency and in Vitro Release Study

The incorporation efficiency of SNL during the preparation of HA/SNL aggregates was determined as follows: 1 mL of HA/SNL aggregates solution was centrifuged at 15,000× *g* for 1 h, and the amount of SNL in the supernatant was measured by high-performance liquid chromatography (HPLC, Agilent 1100 series, Santa Clara, CA, USA). The HPLA analysis was performed using a C-18 column (250 mm × 4.6 mm), a mobile phase of 50 % methanol, a flow rate of 0.5 mL/min, and a detection wavelength of 230 nm. A standard concentration curve SNL was generated for the determination of the SNL content in the aggregates. The incorporation efficiency was calculated using the formula:

Incorporation efficiency = [(Total amount of SNL − Free amount of SNL)/Total amount of SNL] × 100%(1)

The release of SNL from HA/SNA aggregates was investigated using a method similar to abovementioned ones and each measurement was repeated three times. The release of SNL was calculated using:

*In vitro* release (%) = [Total amount of SNL − Residue of SNL]/Total amount of SNL] × 100% (2)

### 3.3. Cell Culture and HA/SNL Aggregates Treatment

A carcinoma cell line A549 was utilized in this study. A549 cells were suspended at a final concentration of 4 × 10^3^ cells/mL and seeded in 96-well plates. Various SNL concentrations (25, 50, and 75 µg/mL) and HA/SNL aggregates (25 and 50 µg/mL SNL) solutions were added to each well in triplicates. After 24 and 48 h of incubation, cell viability was determined through a 3-(4,5-dimethylthiazol-2-yl)-2,5-diphenyltetrazolium bromide (MTT) assay. After exposure to the aforementioned SNL and HA/SNL aggregate treatments at a predetermined time interval, 20 mL of MTT solution was added and the cells were incubated for an additional 3 h. The formazan precipitate was dissolved in 200 mL of DMSO, and the solution was vigorously mixed to dissolve the reacted dye. The absorbance of each well was read on a multiplate reader (Thermo Scientific, Waltham, MA, USA) at 570 nm; wells filled only with culture medium were used for calibration. Cells grown in a medium containing an equivalent amount of DMSO served as control. The cell viability was also examined using a live/dead cell assay (Invitrogen, Cambridge, UK).

### 3.4. Flow Cytometric Analysis of Apoptosis

We examined the anticancer activities of SNL and HA/SNL aggregates in A549 cells through annexin V-fluorescein isothiocyanate (FITC)/propidium iodide (PI) staining for an apoptosis assay and a JC-1 (5,5′,6,6′-tetrachloro-1,1′,3,3′-tetraethylbenzimidazolocarbocyanine iodide) assay to measure differences in the mitochondrial membrane potential. A549 cells (2 × 10^5^ cells/mL) were seeded in T25 culture flasks and incubated for 24 h at 37 °C. The culture media was replaced with 2.85 mL of fresh media, and 0.15 mL of HA nanoparticles and HA/SNL aggregates were added to the flask and incubated for 24 h. The apoptosis assay for A549 cells was performed according to manufacturer instructions by using a staining kit containing FITC-conjugated annexin V/PI (BD Biosciences, San Diego, CA, USA). Briefly, the cells were detached from the culture flask through trypsinization and washed several times with phosphate-buffered saline (PBS). Cell pellets were suspended in 1× binding buffer and stained with 5 µL of annexin V-FITC and 10 µL of PI for 15 min. The cells were subjected to fluorescence-activated cell sorting analysis by using a flow cytometer (BD Accuri C6) [13]. Apoptosis-associated changes in the mitochondrial membrane potential (ΔΨm) were also analyzed using a cationic lipophilic fluorescent probe (JC-1; BD Biosciences). A549 cells were treated with the aforementioned experimental solutions containing HA nanoparticle and HA/SNL aggregates. The JC-1 assay was performed according to the manufacturer protocol for measuring the ΔΨm through flow cytometry.

### 3.5. Cell Cycle Analysis

Cell cycle analyses were performed after culturing A549 cells for 24 h. The cells were harvested, fixed with 70% cold ethanol, and stored at −20 °C for 1 h. They were washed twice with PBS and incubated with phosphate–citrate buffer and 0.1% triton X-100 on a shaking water bath (37 °C, 40 rpm) for 30 min. The cells were washed again with PBS prior to staining with 0.5 mL of PI/RNase staining buffer (BD Biosciences) for 15 min at room temperature. The cells were analyzed through flow cytometry. Cell cycle distribution was calculated from 10,000 cells by using ModFit LT software (Topsham, ME, USA).

### 3.6. Western Blotting Analysis 

Western blot analyses were performed for identifying specific, intracellular apoptosis-related proteins. The concentrations of protein were determined by BCA assay according to the manufacturer’s instructions. 10% SDS-PAGE (SDS-polyacrylamide gel electrophoresis) was used to run and separate the protein samples. A549 cells were treated with SNL or HA/SNL aggregates for 12 h and washed twice with cold-PBS buffer followed by lysis using a cell lysis buffer (50 mM Tris (pH 8.0), 120 mM NaCl, 50 mM NaF, 0.5% NP-40, and 1 mM PMSF). The cell lysates were collected and centrifuged at 12,000 rpm at 4 °C for 30 min. Protein concentration was determined using a Bradford protein assay kit (Bio-Rad Laboratories Inc., Hercules, CA, USA). An equal amount of proteins (100 µg) was loaded, separated through sodium dodecyl sulfate polyacrylamide gel electrophoresis, and transferred to a nitrocellulose membrane. After blocking with 5% skim milk, the membrane was incubated overnight at 4 °C with a solution containing primary antibodies. After washing three times in Tris-buffered saline with 0.1% Tween-20, the membrane was incubated with a solution containing horseradish peroxidase-conjugated secondary antibodies (Santa Cruz Biotechnology, Santa Cruz, CA, USA) for 2 h at room temperature. The antibody reactions were visualized using an enhanced chemiluminescence detection system (Thermo Scientific Pierce Protein Research Products, Rockford, IL, USA).

### 3.7. Wound Healing Assay

The antimigratory effects of SNL and HA/SNL aggregates on A549 cells were examined through a wound healing assay. A wound was created in a cell monolayer using a pipette tip, and the assay was performed in a multiwall plate. The monolayers recovered and healed the wound, which was observed for 24 h. The wound healed as is typical, with cells polarizing toward the edge of the wound, initiating protrusion, migrating, proliferating, and closing the wound. The progression of these events was monitored by manual imaging of the sample at fixed timepoints.

### 3.8. Statistical Analysis

The data are presented as the mean ± standard deviation (SD). Each result was analyzed using an unpaired Student *t* test and the SPSS program, version 17.0 (SPSS Inc., Chicago, IL, USA). *p* < 0.05 was considered significant. Significant differences between the DMSO control and experimental groups with SNL and HA/SNL aggregates are indicated using asterisks (*). (* *p* < 0.05, ** *p* < 0.01, *** *p* < 0.001).

## 4. Conclusions

The results from the present study revealed the cytotoxic, anti-proliferate and apoptosis-inducing effects exerted by marine sponge extracted SNL, and HA/SNL aggregates on A549 lung cancer cells. HA nanoparticle aggregation significantly augmented the anti-migratory and apoptosis-inducing effects compared with pure SNL, suggesting that nanoparticle-based therapeutics play an important role in improving anti-cancer effects. With the evidences from flow cytometric analysis, JC-1 staining and western blot, the mechanism underlying the HA/SNL aggregate-induced A549 cell apoptosis was mitochondria-related. In addition, SNL-conjugated nanoparticles are favorable for reducing prescribed drug doses and achieving more effective therapeutic results. The results of enhanced cytotoxicity with low prescribed drug doses using nanoparticle aggregates offer distinct advantages for treating cancer in the future.

## Figures and Tables

**Table 1:** Apoptotic levels of A549 cells treated with DMSO, SNL, and HA/SNL aggregates.

Conditions	Early Apoptosis	Late Apoptosis
DMSO	0.8%	0.1%
SNL 25 μg/mL	1.5%	0.6%
SNL 50 μg/mL	2.4%	0.9%
SNL 75 μg/mL	12.8%	0.1%
HA/SNL 25	5.3%	1.4%
HA/SNL 50	43.7%	6.4%

**Table 2:** Distribution of A549 cell cycles treated with DMSO, SNL, and HA/SNL aggregates.

Conditions	Sub G1	G1	S	G2/M
DMSO	0.1%	58.7%	22.4%	20.8%
SNL 25 μg/mL	1.9%	59.6%	19.6%	21.3%
SNL 50 μg/mL	3.4%	47.2%	16.4%	34.8%
SNL 75 μg/mL	3.5%	55.5%	13.4%	30.6%
HA/SNL 25	7.4%	55.0%	11.4%	28.8%
HA/SNL 50	9.2%	55.2%	13.1%	25.6%
